# 556 Entering Into a Helping Relationship: A Novel Approach to Burn Center Staff Education/Orientation

**DOI:** 10.1093/jbcr/irac012.184

**Published:** 2022-03-23

**Authors:** Kristen C Quinn, Drew Schnebly, P J Gravis, Giavonni M Lewis, Callie M Thompson

**Affiliations:** University of Utah Health, SLC, Utah; University of Utah Burn Center, Salt Lake City, Utah; University of Utah Health, Salt Lake City, Utah; University of Utah Burn Center, Salt Lake City, Utah; University of Utah Health, Salt Lake City, Utah

## Abstract

**Introduction:**

In the past, our regional burn center’s staff orientation was a robust overview of the interdisciplinary team’s dynamic approach to patient care. 2020 illuminated gaps in our staff orientation and education process, along with seeing increased levels of staff turnover. The need to truly prepare staff to do the work of caring for burn injured patients while also caring for themselves was identified.

A novel approach to orientation was developed; this included the development of a four hour workshop designed to increase awareness of vicarious trauma, the neurobiology of trauma and its impacts on the care we deliver, our home life and our emotional wellbeing. Social taboos surrounding self-care were addressed. A deep dive into trauma informed care formed the foundation of the workshop. Participants were given tools they can use at work, home, for patients and themselves to increase self-awareness regarding vicarious trauma and implement change in their daily lives.

**Methods:**

Registration was prioritized for new burn staff and open to veteran members of the team. Material was presented via a variety of modalities: art, team build exercise, didactic lecture and discussion. This course will be offered bi-annually. A post course survey was completed; see attached evaluation and graphed likert scale.

**Results:**

The 17 participants included nurses, CNS’s and paramedics;15 from the burn center and 2 from the emergency department. Evaluations were overwhelming positive (see graph). Comments included “it really helped me understand more of the psychological part of my own care”, “it taught me to look at my family and my patient’s family through their world view”, “this was the most important class I’ve ever been given as a nurse”.

**Conclusions:**

Given the success of this novel approach to nursing education/orientation, we are looking for additional pathways to offer this workshop. Follow up discussions with participants have indicated sustained benefits from this program. We are exploring options to measure these outcomes and impacts on longevity and retention.

